# Kidney transplantation in patients with ANCA-associated vasculitis is associated with a high incidence of post-transplant cancer

**DOI:** 10.1007/s40620-024-01951-6

**Published:** 2024-06-04

**Authors:** Alessandro Perna, Mariarosaria Campise, Carlo Maria Alfieri, Anna Regalia, Donata Cresseri, Maria Teresa Gandolfo, Giuseppe Castellano

**Affiliations:** 1 Department of Translational Medical Sciences, University of Campania ‘L. Vanvitelli’, Naples, Italy; 2 Department of Nephrology, Dialysis and Renal Transplantation, Fondazione IRCCS Cà Granda Ospedale Maggiore Policlinico, Milan, Italy; 3 Department of Clinical Sciences and Community Health, University of Milan, Via Commenda 15, 20122 Milan, Italy; 4 Nephrology, IRCCS Ospedale Policlinico San Martino, 16132 Genoa, Italy

**Keywords:** Kidney transplant, ANCA, Vasculitis, Cancer, Transplant complications

## Abstract

**Background:**

Antineutrophil cytoplasmic antibody (ANCA)-associated vasculitis (AAV) is a rare disease with limited data on outcomes after transplantation.

**Methods:**

In this single-center retrospective cohort study, we describe the outcomes of kidney transplant patients with AAV transplanted at our institute from February 2006 to January 2022.

**Results:**

We identified 9 patients among 1026 with a pre-transplant diagnosis of AAV; all patients had received previous treatment with cyclophosphamide. Maintenance immunosuppression after transplantation was tacrolimus-based in 89% of the patients. At the end of a mean follow-up of 132 ± 61.1 months after transplantation, only one case of extrarenal vasculitis relapse was observed. The relapse rate was 0.01 per patient per year, which is comparable to that reported in the literature. However, seven patients were diagnosed with cancer after a mean follow-up of 81.4 months after transplantation; six had skin cancer and three had renal cell carcinoma (RCC) of the native kidneys (cumulative incidence of 78%). One patient died from metastatic squamous cell carcinoma.

**Conclusion:**

In this study, we found a noticeable decrease in disease relapse (1 relapse in the present cohort vs 7 relapses in 19 patients in the previous cohort) in kidney transplant patients with AAV compared with previous data from our group (December 1987–January 2006). Conversely, we found a high incidence of post-transplant cancer. This result could be attributed to reduced immunosurveillance due to immunosuppression therapy before and after transplantation. Therefore, constant cancer early diagnosis and prevention is mandatory during the post-transplant follow-up of AAV patients.

**Graphical abstract:**

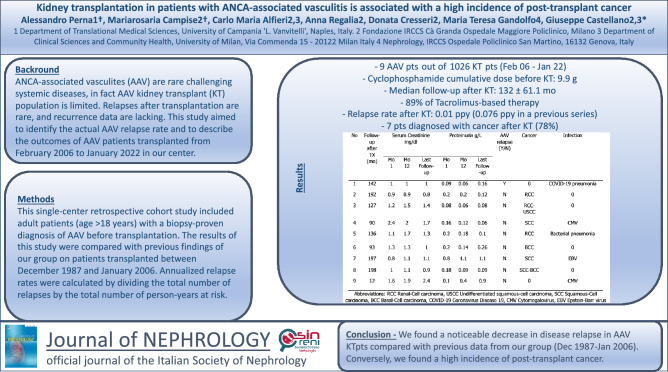

## Introduction

Antineutrophil cytoplasmic antibody (ANCA)-associated vasculitides are challenging systemic diseases [1]. Renal involvement with glomerular capillary damage is frequent [2]. AAV is a rare disease with a worldwide reported annual incidence of 1.2–2.0 cases per 100,000 individuals and a prevalence of 4.6–18.4 cases per 100,000 individuals [3]. Incidence increases with age, with a peak at 60–70 years of age; there is a slight male preponderance [4].

Kidney biopsy shows the pathological hallmark of ANCA-associated glomerulonnephritis (GN), which is a necrotizing and/or crescentic GN without significant immune complex deposition that is detectable using immunofluorescence or electron microscopy [5]. Kidney transplantation (KTx) is the treatment of choice for patients with AAV and end-stage kidney disease (ESKD) [6]. AAV relapses after transplantation are rare, and recurrence data are lacking. Relapse rates ranging from as low as 0.006 to 0.1 per patient per year [7] can potentially lead to transplant loss. Therefore, prompt diagnosis followed by appropriate treatment is of pivotal importance. Still, standardized treatment protocols and adequate follow-up guidelines are not available. Regarding AAV activity after transplantation, close laboratory monitoring of serum creatinine, ANCA quantification test, and 24 h proteinuria and urinary sediment should be performed. Treatment of AAV recurrence is similar in both transplanted and naive patients; remission can be achieved using methylprednisolone pulses followed by 1 mg/kg/day of oral prednisone associated with cyclophosphamide or rituximab according to the chosen protocol [8].

In this retrospective study, we evaluated the actual AAV relapse rate after kidney transplantation among patients who underwent transplantation in our hospital between February 2006 and January 2022. The results were compared with previous findings by our group on patients transplanted between December 1987 and January 2006 [9].

## Methods

### Study design

In this single-center retrospective cohort study, we analyzed data from the medical records of adult patients transplanted at our transplant Center between February 2006 and January 2022 and followed-up at the Nephrology Unit of the same hospital: Fondazione IRCCS Cà Granda Ospedale Policlinico in Milan.

### Patient characteristics

All adult patients (age > 18 years) with a biopsy-proven diagnosis of AAV before transplantation were included in the study. AAV diagnosis was established according to the Chapel Hill Classification and European Alliance of Associations for Rheumatology (EULAR) criteria.

### Relapse rate

Annualized relapse rates were calculated by dividing the total number of relapses by the total number of person-years at risk.

All procedures performed in this study involving human participants were in accordance with the ethical standards of the institutional and/or national research committee and with the 1964 Declaration of Helsinki and the Declaration of Istanbul and its later amendments or comparable ethical standards. Informed consent was obtained from all participants.

## Results

One thousand and twenty-six (1026) patients underwent KTx at our center between February 2006 and January 2022. Nine patients met the inclusion criteria. Six of the nine patients were granulomatosis with polyangiitis (GPA) proteinase 3 (PR3)-ANCA positive. Three patients were microscopic polyangiitis (MPA) myeloperoxidase (MPO)-ANCA positive. The patients’ pre-transplant characteristics are presented in Table 1.

**Table 1:** Patients’ pre-transplant characteristics

No	Diagnosis	ANCA	Organ involvement	Treatment	Cumulative CTX dose (g)	Pre-Tx relapse	Relapse treatment		Dialysis duration (mo)		Time between diagnosis and dialysis (mo)	Biopsy findings
1	GPA	P-ANCA PR3	Kidney/lung	CS/CTX/AZA/PLF	4.5	No	0		6		206	CGN
2	GPA	C-ANCA PR3	Kidney/lung	CS/CTX	14	Yes	CS/CTX		144		36	CGN
3	GPA	C-ANCA PR3	Kidney/lung	CS/CTX/AZA	9	Yes	NA		65		48	CGN
4	GPA	C-ANCA PR3	Kidney/joints	HD	12	Yes	CS/CTX		161		0	CGN
5	GPA	C-ANCA PR3	Kidney/lung	CS/CTX	13.5	Yes	CS/CTX		120		24	CGN
6	GPA	C-ANCA PR3	Kidney/lung	CS/CTX/AZA/PLF	12	No	0		60		0	CGN
7	MPA	P-ANCA MPO	Kidney	CS/CTX/HD	9	No	0		17		78	CGN
8	MPA	P-ANCA MPO	Kidney/joints	CS/CTX	5.5	No	0		24		9	CGN
9	GPA	C-ANCA PR3	Kidney/joints	CS/CTX/PLF	10	No	0		0		0	CGN

*mo* months, *GPA* granulomatosis with polyangiitis, *MPA* microscopic polyangiitis, *PR3* anti-proteinase 3 antibodies, *MPO* anti-myeloperoxidase, *CS* steroids, *CTX* cyclophosphamide, *AZA* azathioprine, *PLF* plasmapheresis, *HD* hemodialysis, *CGN* crescentic glomerulonephritis, *NA* not available

### AAV treatment before transplantation

At diagnosis, 8 patients were treated with methylprednisolone pulses (500–1000 mg daily for 3 days) and intravenous cyclophosphamide (15 mg/kg) every 2–3 weeks for 3–6 months, plus prednisone at the initial dose of 1 mg/kg per day tapered to 5–10 mg/day. As maintenance therapy, azathioprine: (range 2 mg–0.5 mg/kg/day) or oral cyclophosphamide (2 mg/kg/day) were added to the low prednisone dose for 12–18 months. The cumulative dose of cyclophosphamide is reported in Table 1. Three patients were also treated with plasmapheresis. One patient did not receive any treatment because of rapidly progressive glomerulonephritis leading to renal failure requiring immediate dialysis. Before transplantation, 4 of 9 (44.4%) patients experienced a relapse with lung involvement and were treated with corticosteroids and cyclophosphamide (Table 1).

### Patients’ characteristics at kidney transplantation

At transplantation, the mean age was 55 ± 10.5 years, 4 patients were male. Seven patients (77.8%) received a deceased donor transplant and two received a living donor kidney: one was a pre-emptive transplant. Median time on dialysis was 74.6 [161–24] months. At the time of transplantation, all patients were in clinical remission, ANCAs were positive in 4 of them (44.4%).

### Immunosuppressive protocol

Induction therapy included basiliximab (20 mg day 0–4) and methylprednisolone (500 mg on day 0, 125 mg day 1–2) according to our internal protocol in all patients. Maintenance therapy was mycophenolate mofetil (2 g per day tapered to 1 at the end of the first year), prednisone (20 mg per day tapered to 5 mg per day at day 60), and tacrolimus (0.1 mg/kg bid) in 8 patients (88.9%). Cyclosporine (3 mg/kg per day tapered to 1.5 mg per day at month 2), prednisone, and everolimus (1 mg bid) were used in one patient. In two patients, mycophenolate mofetil was withdrawn; it was replaced with Azathioprine in one patient because of a pregnancy plan 31 months after transplant, while in the second patient, no other drug was introduced because of an Epstein-Barr virus infection (Table 2).

**Table 2:** Patients’ characteristics and immunosuppression treatment at kidney transplantation

No	Gender	Age	TX Donor	Pre-emptive TX (Y/N)	Induction Agents	Maintenance therapy	ANCA status ( ±)
1	F	33	LD	N	Basiliximab + Methylprednisolone	P-TCR-MMF shift to P-TCR-AZA after 31 mo	–
2	F	57	DD	N	Basiliximab + Methylprednisolone	P-EVR-CyA	–
3	M	58	DD	N	Basiliximab + Methylprednisolone	P-TCR-MMF	–
4	M	66	DD	N	Basiliximab + Methylprednisolone	P-TCR-MMF	+
5	F	46	DD	N	Basiliximab + Methylprednisolone	P-TCR-MMF	+
6	M	50	DD	N	Basiliximab + Methylprednisolone	P-TCR-MMF	–
7	F	62	LD	N	Basiliximab + Methylprednisolone	P-TCR-MMF after 37 mo stop MMF	–
8	F	59	DD	N	Basiliximab + Methylprednisolone	P-TCR-MMF	+
9	M	65	DD	Y	Basiliximab + Methylprednisolone	P-TCR-MMF	+

*TX* transplantation, *mo* months, *LD* living donor, *DD* deceased donor, *P* prednisone, *TCR* tacrolimus, *AZA* azathioprine, *MMF* mycophenolate mofetil, *EVR* everolimus, *CyA* Cyclosporine

### Outcome analysis

During follow-up no AAV relapse with renal involvement was observed. One patient with granulomatosis with polyangiitis had a biopsy-confirmed extrarenal recurrence 53 months after transplantation (relapse rate 0.011 per patient per year). The recurrence affected the paranasal sinuses (biopsied), and the eyes, causing visual acuity reduction and exophthalmos. ANCA was negative throughout the relapse. The patient was treated with methylprednisolone pulses: 500 mg/day for three consecutive days, followed by oral prednisone 0.5 mg/kg/day. Two Rituximab doses of 1 g each were also administered 15 days apart after the methylprednisolone pulses, while azathioprine was suspended. The patient showed rapid improvement in symptoms and achieved complete remission.

The mean follow-up period was 132 ± 61.1 months.

One patient died 127 months after transplantation of metastatic squamous cell carcinoma.

During the observation period, no significant variation in creatinine or proteinuria was detected: mean serum creatinine 1.38 ± 0.4 mg/dl (at month 12), and mean creatinine at the end of the observation period was 1.29 ± 0.5 mg/dl. Mean proteinuria was 0.261 ± 0.3 g/L (at month 12) and 0.318 ± 0.4 mg/L at the end of follow-up. No patient experienced acute rejection episodes. Two patients underwent kidney biopsy for rapid and progressive worsening of renal function. In one patient who had an almost doubling of creatinine (1.5 to 2.6 mg/dl) without significant proteinuria, negative urinary sediment, and negative ANCA, the renal biopsy result was consistent with calcineurin inhibitor toxicity. Tacrolimus dosage was reduced, and creatinine levels returned to baseline values. In the second patient, because of persistent, incomplete renal recovery (creatinine 2.39 mg/dl at month 6), a renal biopsy was performed and showed diffuse interstitial fibrosis and diffuse arteriosclerotic damage attributable to the donor (Table 3).

**Table 3:** Post-transplant follow-up and outcome

No	Post Tx F-up (mo)	Serum Creatinine (mg/dl)			Proteinuria g/L				AAV relapse (Y/N)	TX biopsy (Y/N)	Cancer	CV disease	Diabetes (Y/N)	Infection
		Mo 1	Mo 12	Last Follow-up	Mo 1	Mo 12	Last Follow-up							
1	142	1	1	1	0.09	0.06		0.16	Y	N	0	0	N	COVID-19 pneumonia
2	192	0.9	0.9	0.8	0.2	0.2		0.12	N	N	RCC	HTN	N	0
3	127	1.2	1.5	1.4	0.08	0.06		0.08	N	N	RCC-USCC	HTN	N	0
4	90	2.4	2	1.7	0.16	0.12		0.06	N	N	SCC	HTN	N	CMV
5	136	1.1	1.7	1.3	0.2	0.18		0.1	N	Y	RCC	HTN AF	N	Bacterial pneumonia
6	93	1.3	1.3	1	0.2	0.14		0.26	N	N	BCC	HTN	Y	0
7	197	0.8	1.1	1.1	0.8	1.1		1.1	N	N	SCC	HTN	N	EBV
8	198	1	1.1	0.9	0.18	0.09		0.09	N	N	SCC-BCC	HTN	N	0
9	12	1.6	1.9	2.4	0.1	0.4		0.9	N	Y	0	HTN	N	CMV

*F-up* follow-up, *mo* months, *RCC* renal-cell carcinoma, *USCC* undifferentiated squamous-cell carcinoma, *SCC* squamous-cell carcinoma, *BCC* basal-cell carcinoma, *CV* cardiovascular, *HTN* hypertension, *AF* atrial fibrillation, *CMV* cytomegalovirus, *EBV* Epstein-Barr virus

Finally, we carried out a narrative comparison between a previous series published by our group (Table 4) including patients transplanted between December 1987 and January 2006 (cohort 1: 1225 patients) [9] and the most recent cohort including patients transplanted between February 2006 and January 2022 (cohort 2: 1026 patients). In the first cohort, 7 relapses in 19 patients were observed: relapse rate: 0.076 per patient per year. After relapse, 4 patients experienced an acute rejection episode, and three of them developed rapidly progressive renal insufficiency. In the present cohort, only one extrarenal relapse in 9 patients was observed (relapse rate of 0.011 per patient per year), and no acute rejection episodes were diagnosed. Forty-seven percent of cohort 1 patients were treated with cyclosporine-based maintenance immunosuppression, whereas tacrolimus-based immunosuppression was used in 89% of cohort 2 patients.

**Table 4:** Comparison of the two cohorts

	12/1987—01/2006 Cohort 1	02/2006—01/2022 Cohort 2
KTx	1225	1026
AAV patients	19	9
Age (mean ± SD)	46.6 ± 12.7	55 ± 10.5
Follow-up in months (mean ± SD)	58 ± 57	132 ± 61.1
Relapse (%)	7 (37%)	1 (11%)
Relapse rate (ppy)	0.076	0.011
Acute rejection episodes (%)	5 (26%)	0
Deaths (%)	2 (11%)	1 (5%)
Graft loss (%)	2 (11%)	0
Anti-rejection therapy CyA (%)/TCR (%)	9 (47%) / 10 (53%)	1 (11%) / 8 (89%)
N° of patients with cancer (%)	NA	7 (78%)
N° patients with Infection and hospitalization (%)	14 (74%)	2 (22%)

*KTx* kidney transplant, *CyA* cyclosporine, *TCR* tacrolimus, *NA* not available

### Post-transplant complications

The most relevant complications during follow-up were infections, cardiovascular complications, and cancer.

The cumulative incidence of infection was 55%. Two patients had cytomegalovirus infection. One patient became infected (D + /R–) 5 months after transplantation following the prophylactic treatment period. The second patient experienced cytomegalovirus reactivation (D + /R +) 17 days after transplantation. Another patient had Epstein– Barr virus infection that was treated with a reduction in immunosuppressive therapy (mycophenolate mofetil withdrawal) and became DNA negative.

During follow-up, two patients were hospitalized: one for SARS-CoV-2 pneumonia and the other for community-acquired pneumonia, both of which resolved without any sequelae.

No major cardiovascular events were observed during follow-up; 88.9% of patients were hypertensive and on treatment with at least two drugs. One patient initiated oral anticoagulant treatment for atrial arrhythmia, and one patient (11.1%) developed insulin-dependent diabetes.

**Fig. 1:**
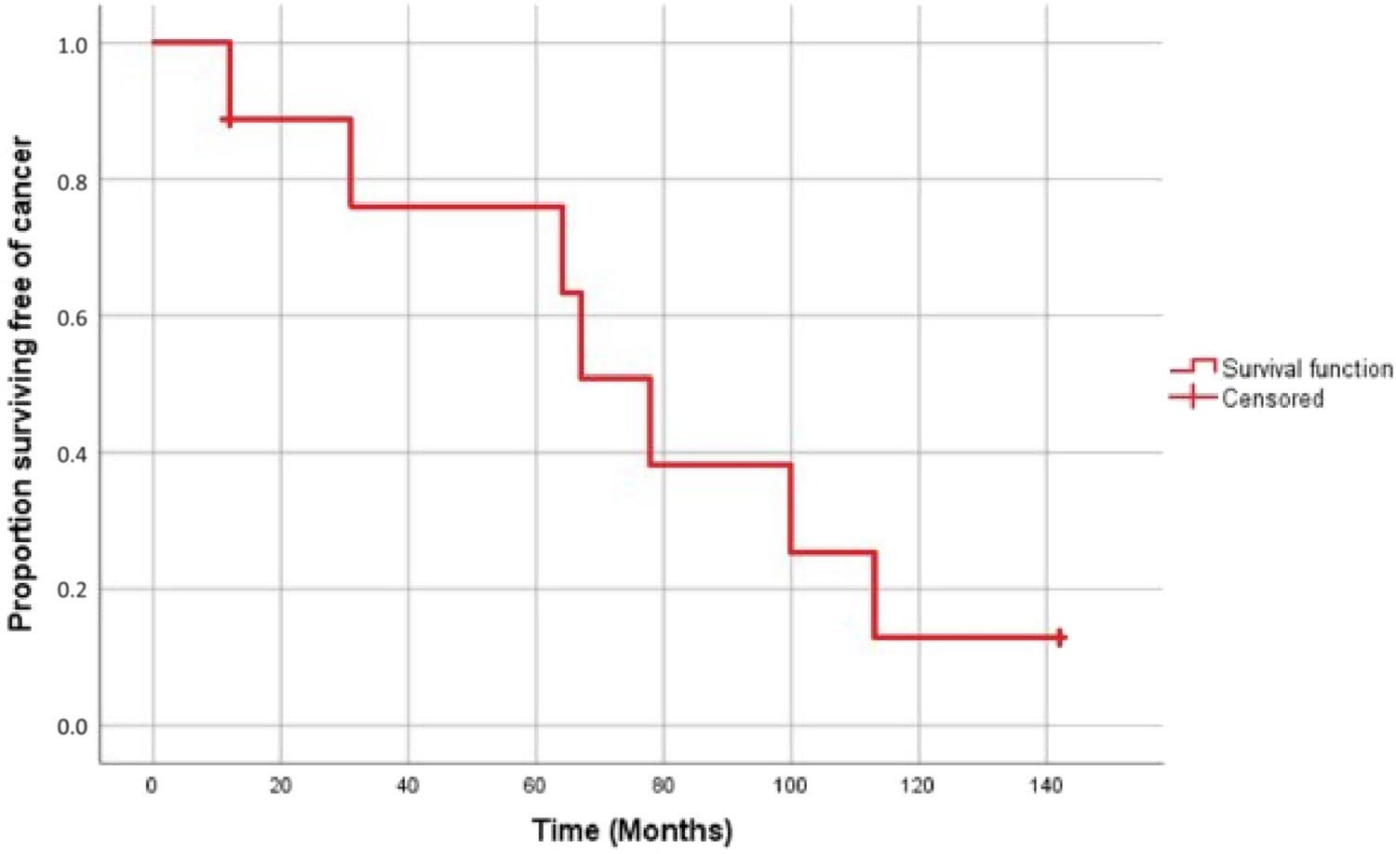
Curve for the cumulative risk of cancer

Interestingly, seven patients were diagnosed with cancer (cumulative incidence 77.8%). Two patients had squamous cell carcinoma, one had basal cell carcinoma, and one had both. Two patients had renal cell carcinoma (RCC) of the native kidneys. One patient had both undifferentiated squamous-cell carcinoma and RCC, and died of metastatic skin cancer. The curve for the cumulative risk of cancer is shown in Fig. 1.

## Discussion

AAVs are challenging glomerular diseases at risk of relapse [10] after transplantation and can potentially lead to graft loss. However, recent reports showing a decreasing relapse risk, confirm that kidney transplantation remains the best treatment for patients with AAV provided that constant post-transplant monitoring is performed [11].

The first relevant finding of our study is the increased incidence of cancer after kidney transplantation among patients with AAV who received treatment with cyclophosphamide before transplantation. Non-melanoma skin cancer and native kidney cancer were diagnosed with a cumulative incidence of 78% after a mean follow-up of 81.4 months after transplantation. This result is in agreement with a recent study that showed an increased incidence of malignancy after five years of immunosuppression for the treatment of AAV [12], and a further increase after kidney transplantation as reported in a Norwegian registry-based study with a mean follow-up period of 86 months (SIR = 2,12,95% CI 1,01–4,4) [13].

All our patients received treatment with cyclophosphamide before transplantation, with a mean cumulative dose of 9.9 g. It is well known that kidney transplanted patients are at increased risk of developing cancer compared with the general population because of continuous immunosuppressive therapy [14, 15]. This concept is also applicable to patients with AAV. Immunosuppressive drugs cause impaired immunosurveillance with direct and indirect effects, favoring the degeneration of inflamed tissues [16]. Several studies have shown that cyclophosphamide plays a central role in the promotion of carcinogenesis [17, 18]. We observed an increased incidence of non-melanoma skin cancer and native kidney cancer. In such a small series, no correlation was found with the causative agent. However, the growing use of rituximab compared with cyclophosphamide could reduce such risk [19]. Treatment with rituximab and glucocorticoids is not inferior to the standard cyclophosphamide regimen when used as induction treatment [20], although it is not associated with a reduction in early severe adverse events like infection and cancer diagnosis [21]. In the long term, the malignancy risk in patients with AAV is lower in rituximab-treated patients than in those treated with cyclophosphamide [22]. Rituximab should be chosen as the first-line therapy with corticosteroids to induce remission of severe AAVs, especially if cyclophosphamide represents a contraindication (relapse after cyclophosphamide treatment, women of childbearing age, etc.) [23].

The second relevant finding of this series comes from the comparison with our historical cohort: we found a significant decrease in the relapse rate (0.076 vs 0.011).

In cohort 1, we observed seven relapses in 19 pts [9]. Four patients relapsed within 3 months after transplantation. Relapse was associated with an acute rejection episode and rapidly progressive renal insufficiency leading to graft loss in one patient. In this series, we observed only one extrarenal relapse among nine patients, which was not followed by an acute rejection episode. The relapse risk dropped from 0.076 to 0.011, similar to that reported in the literature [24]. Early diagnosis and treatment of suspected relapse may account for these results. Furthermore, the increased use of tacrolimus instead of cyclosporine as maintenance therapy [25], which reduces the incidence of acute rejection by providing stronger immunosuppression, also reduced the risk of relapse. Indeed, only one patient in this series, i.e., the one with the longest transplant vintage, was treated with cyclosporine. Low recurrence results rely on new and more powerful immunosuppressive therapies and on remission attained before listing for transplantation. Stronger immunosuppression, however, exposes all patients to a greater risk of malignancy.

The last finding was the reduced incidence of infections requiring hospitalization: 22% vs 74% in our previous study. This finding is consistent with more severe disease activity at diagnosis and after transplantation. In contrast to what was reported in the literature, we could not relate this result with patients’ age since cohort 1 patients were younger than those in cohort 2 [26].

In conclusion, our results, albeit based on a small sample-size, show a consistent decrease in AAV relapse after transplantation and an increased incidence of post-transplant cancer, the latter attributable to the use of more powerful immunosuppressants. Therefore, continuous and careful cancer surveillance during post-transplant follow-up of these patients is recommended. Future multicenter studies are needed to confirm our results and improve the treatment of this aggressive kidney disease.

## Data Availability

The datasets generated and analyzed during the current study are available from the corresponding author on reasonable request.
